# Giant Postpartum Ovarian Mixed Germ Cell Tumor Containing Mature Cystic Teratoma and Yolk Sac Tumor: A Case Report With Multimodality Imaging and Clinicopathological Correlation

**DOI:** 10.7759/cureus.116839

**Published:** 2026-09-24

**Authors:** Kabelo Molatedi, Mikail Amod, Nhlanhla Mphuthi, Glad Masingi, Godfrey Letlalo

**Affiliations:** 1 Department of Radiology, Robert Mangaliso Sobukwe Hospital, Kimberley, ZAF; 2 Department of Orthopedic Surgery, Frere Tertiary Hospital, East London, ZAF; 3 Department of Emergency Medicine, Leratong Hospital, Krugersdorp, ZAF; 4 Department of Hematology, National Health Laboratory Service, Johannesburg, ZAF; 5 Department of Family Medicine, Johannesburg Health District, Johannesburg, ZAF

**Keywords:** alpha-fetoprotein, case report, computed tomography, mature cystic teratoma, mixed germ cell tumor, ovarian neoplasm, postpartum, yolk sac tumor

## Abstract

Ovarian mixed germ cell tumors containing mature cystic teratoma and yolk sac tumor (YST) can be difficult to characterize on imaging, particularly when the mass is very large and heterogeneous. A 20-year-old woman presented approximately six months after vaginal delivery with progressive abdominal distension, right lower quadrant pain, weight loss, and amenorrhea. CT demonstrated a giant complex abdominopelvic mass, while hepatic abnormalities were reported as suspicious for metastases. The initial radiological differential included immature ovarian teratoma and choriocarcinoma. Serum alpha-fetoprotein (AFP) was >60,500 µg/L, whereas beta-human chorionic gonadotropin (β-hCG) was 2 IU/L. Following right salpingo-oophorectomy, the specimen weighed 3,697 g and measured 25 × 20.6 × 13.1 cm. Histopathology demonstrated a mixed germ cell tumor composed of mature cystic teratoma and YST, with YST accounting for approximately 70% of the tumor volume. No immature teratomatous component was identified. The pathology report assigned a pT1a category to the primary tumor; the overall stage could not be established because the hepatic findings remained unresolved. Postoperative treatment and longitudinal follow-up data were unavailable. This case highlights the value of interpreting imaging alongside serum tumor markers and histopathology. Marked AFP elevation supported a YST component despite the initial imaging differential, while tissue examination established the mature nature of the teratomatous component. Progressive postpartum abdominal enlargement accompanied by pain, weight loss, or a palpable mass warrants investigation for underlying pathology.

## Introduction

Malignant ovarian germ cell tumors (MOGCTs) are rare, accounting for approximately 2.6% of ovarian malignancies, and predominantly affect adolescents and young women [1-4]. They encompass dysgerminoma, yolk sac tumor (YST), embryonal carcinoma, non-gestational choriocarcinoma, immature teratoma, and mixed germ cell tumors [1-3]. Their occurrence during the reproductive years makes accurate classification particularly important because treatment can often incorporate fertility-preserving surgery and platinum-based chemotherapy [2,3].

YST, also termed endodermal sinus tumor, is a malignant non-dysgerminomatous germ cell tumor characterized by rapid growth and a strong association with marked serum alpha-fetoprotein (AFP) elevation. It commonly presents as a large heterogeneous solid-cystic ovarian mass and may contain necrotic or hemorrhagic components, producing substantial imaging overlap with other germ cell tumors, particularly immature teratoma [1,4]. Its aggressive biological behavior and potential for extraovarian spread make timely recognition, staging, and treatment clinically important. Tumor markers and histopathology therefore play central roles in establishing the final diagnosis.

Mixed germ cell tumors contain two or more germ cell tumor components. The coexistence of YST and mature cystic teratoma is uncommon and diagnostically important because a large heterogeneous mass may show teratomatous imaging features while the clinically dominant malignant component is YST; imaging therefore cannot reliably determine the precise histological composition [4,5]. Ovarian YST has also been reported during pregnancy [6]. We describe a giant ovarian mixed germ cell tumor diagnosed approximately six months after delivery in a 20-year-old woman, with a predominant YST component, serum AFP >60,500 µg/L, and radiologically suspicious hepatic lesions. The principal learning objective is to demonstrate how multimodality imaging, serum tumor markers, and histopathology should be integrated when differentiating components of a mixed ovarian germ cell tumor while avoiding overstatement of radiologically suspicious extraovarian disease.

## Case presentation

Clinical presentation

A 20-year-old woman (gravida one, para one) presented approximately six months after an uncomplicated vaginal delivery with progressive abdominal distension that had begun after delivery. Right lower quadrant pain had developed approximately three months before presentation and radiated to the right leg. She also reported approximately four months of amenorrhea, weight loss, and reduced appetite. She smoked but had no documented chronic illnesses, regular medication use, previous surgery, or drug allergies. Detailed antenatal records and the original pregnancy ultrasound examinations were not available to the authors; therefore, the frequency and findings of antenatal ultrasonography and any assessment of the adnexa during pregnancy cannot be reliably reported.

Examination showed a wasted appearance, marked abdominal distension, a large central abdominal mass, and prominent abdominal veins. There was right lower quadrant tenderness. Serial examinations described the mass as mobile and soft or semi-fluctuant. A rapid pregnancy test was negative. An abdominocentesis had been attempted at the referring hospital before transfer.

Imaging findings

Initial pelvic ultrasonography described a mass separate from a small uterus, with a small amount of fluid in the pouch of Douglas; both ovaries were initially reported as normal. Transabdominal ultrasonography described a large, well-circumscribed mass superior to the uterus with homogeneous hypoechoic contents. No original ultrasound images were available for review.

Contrast-enhanced CT demonstrated a giant complex abdominopelvic mass occupying a substantial proportion of the abdominal and pelvic cavities. The recorded radiological differential included immature ovarian teratoma and choriocarcinoma, and hepatic abnormalities were considered suspicious for metastatic disease. A later ultrasound examination described bilateral cystic adnexal masses, severe hepatomegaly, and solid hepatic lesions. These descriptions were reproduced from the available retrospective clinical records. The apparent discrepancy between the initial ultrasound, which reported both ovaries as normal, and the later ultrasound describing bilateral cystic adnexal masses could not be resolved because the original ultrasound images were unavailable for independent review; it is therefore reported as a limitation rather than interpreted as documented interval tumor development.

Representative CT screenshots demonstrate the mass in axial, coronal, and sagittal planes (Figure 1). The available screenshots did not permit confident independent localization or characterization of the reported hepatic lesions, and hepatic metastases were not confirmed histologically. The CT appearance was therefore concerning for an MOGCT, but imaging alone could not establish the precise germ cell subtype.

**Figure 1:**
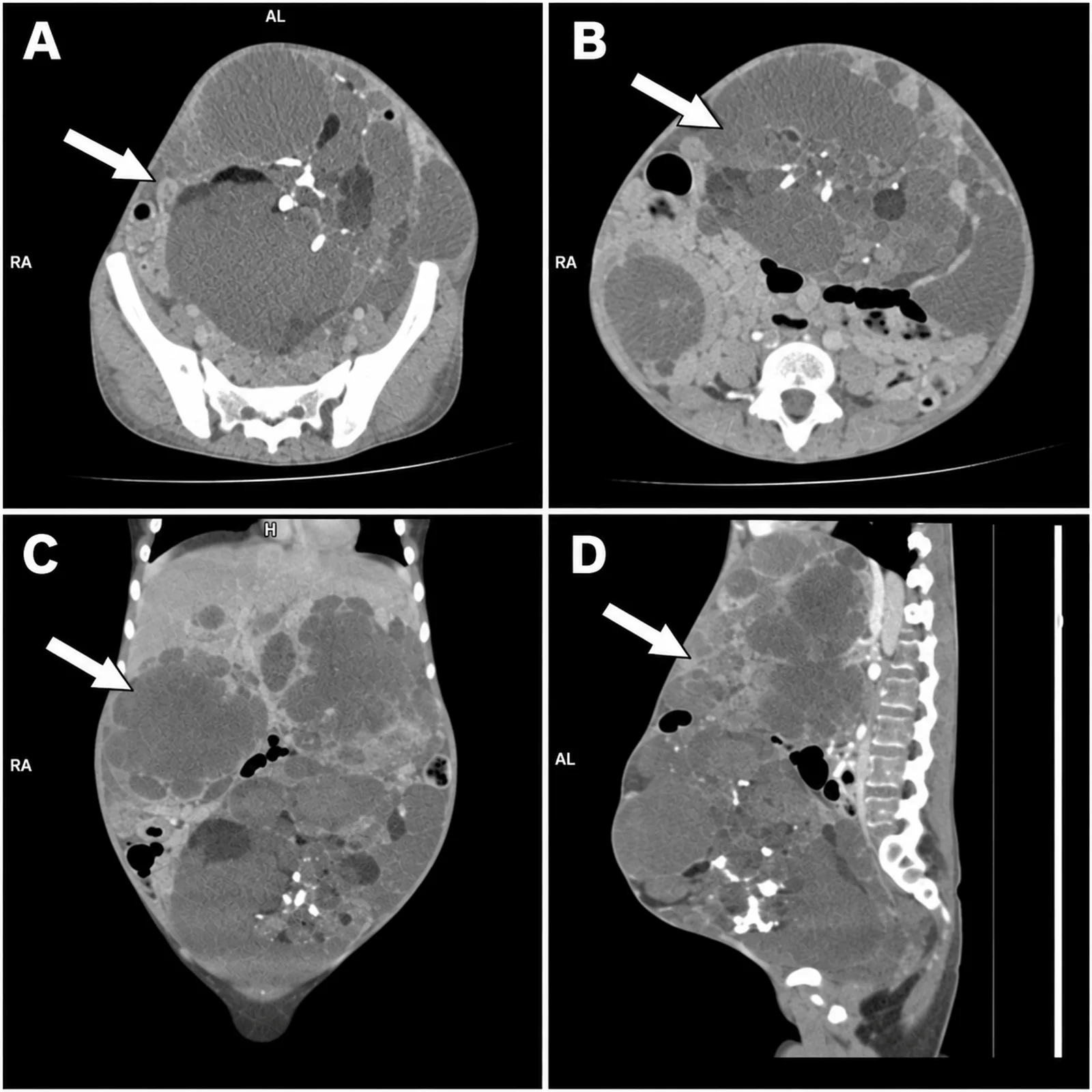
Multiplanar contrast-enhanced CT of the abdominopelvic mass. White arrows identify the heterogeneous mass on axial images (A, B), its inferior component on the coronal reconstruction (C), and its lower abdominal component on the sagittal reconstruction (D). The arrows do not identify hepatic lesions or individual histological components. Images are cropped screenshots; original DICOM data were unavailable.

Tumor markers and laboratory findings

Serum AFP was >60,500 µg/L, beta-human chorionic gonadotropin (β-hCG) was 2 IU/L, CA-125 was 322 U/mL, and CA19-9 was 1,314 U/mL; these tumor marker results were documented on D8 of the available clinical timeline. The marked AFP elevation supported a YST component, whereas the low β-hCG concentration did not support the initial consideration of choriocarcinoma [1,4].

The patient had microcytic anemia, with hemoglobin values of 7.3 g/dL and a documented nadir of 7.1 g/dL. Hemoglobin subsequently increased to 8.5 g/dL after red cell transfusion (documented on D13); a later value was 8.3 g/dL. Exact sampling times were not available for every hemoglobin measurement. Relevant laboratory results and the available laboratory reference intervals are summarized in Table 1. Reference intervals were not available for CA-125 and CA19-9 in the supplied report.

**Table 1:** Tumor markers and selected laboratory investigations. Tumor markers and selected laboratory investigations. Tumor marker measurements (AFP, β-hCG, CA-125, and CA19-9) were documented on D8. A post-transfusion hemoglobin of 8.5 g/dL was documented on D13; exact sampling times were unavailable for some other hemoglobin and laboratory measurements. Results are reproduced from the laboratory values and clinical notes available to the authors. Reference intervals are taken from the National Health Laboratory Service laboratory report where available. Reference intervals for CA-125 and CA19-9 were not shown in the report. AFP: alpha-fetoprotein; β-hCG: beta-human chorionic gonadotropin; CA: carbohydrate antigen; MCV: mean corpuscular volume; WCC: white cell count

Investigation	Result	Unit	Reference range
AFP	>60,500	µg/L	0-7
β-hCG	2	IU/L	≤1 (nonpregnant premenopausal women)
CA-125	322	U/mL	Not reported in laboratory report
CA19-9	1,314	U/mL	Not reported in laboratory report
Hemoglobin	7.3; nadir 7.1; later 8.5 and 8.3	g/dL	11.6-16.4
WCC	14.63	× 10⁹/L	3.90-12.60
MCV	73.6	fL	78.9-98.5
Platelets	481	× 10⁹/L	186-454
Ferritin	722	µg/L	15-150
Serum iron	1.6	µmol/L	9.0-30.4
Transferrin	2.02	g/L	2.50-3.80

Surgical and histopathological findings

After referral from Robert Mangaliso Sobukwe Hospital to the gynecology service at Universitas Academic Hospital, the patient underwent right salpingo-oophorectomy. The specimen comprised a right ovarian mass with an attached fallopian tube and fimbria. It weighed 3,697 g and measured 25 × 20.6 × 13.1 cm. The external surface was smooth and intact, without adhesions, hemorrhage, or papillary structures. The cut surface was nodular, and the report separately described a cystic and solid tumor component measuring 14 × 13.5 × 10.4 cm. The cystic component contained keratin and hair shafts.

Histopathology demonstrated a mixed germ cell tumor comprising mature cystic teratoma and YST. The teratomatous component contained mature skin, adipose tissue, and cartilage. The YST component showed trabecular and tubular architecture, lace-like and microcystic areas, endodermal sinus patterns with Schiller-Duval bodies, and focal solid growth. There was abundant tumor necrosis. YST accounted for approximately 70% of the tumor volume, and no immature teratomatous component was identified.

The YST component stained positively for spalt-like transcription factor 4 (SALL4), placental alkaline phosphatase (PLAP), and cytokeratin AE1/AE3, and negatively for CD117 and CD30. No residual ovarian stroma was identified, and the fallopian tube showed no significant histological abnormality. The pathology report described the tumor as confined to the ovary and assigned a pT1a category.

The pathology report also recorded a clinical history of suspected liver metastases without histological confirmation. The pT1a category is retained as reported for the primary tumor; it does not establish the overall stage. The nature of the hepatic lesions and the overall disease stage therefore remained unresolved in the records reviewed.

Clinical course and follow-up

Initial management included analgesia, red cell transfusion, and supportive care before referral for definitive surgery. Postoperative oncology records from Universitas Academic Hospital were unavailable to the authors. Whether systemic chemotherapy was administered, the postoperative AFP response, the subsequent evolution of the radiologically suspicious hepatic lesions, and long-term disease status could therefore not be established and have not been inferred.

Timeline

Table 2 summarizes the documented clinical course. Day 1 denotes the first recorded presentation, with subsequent events expressed relative to that day.

**Table 2:** Clinical timeline. Day 30 denotes specimen receipt for pathological examination, not a confirmed operation date. AFP: alpha-fetoprotein; β-hCG: beta-human chorionic gonadotropin; CA: carbohydrate antigen; YST: yolk sac tumor

Study day	Clinical event
Day 1	Initial presentation with progressive postpartum abdominal distension, right lower quadrant pain, amenorrhea, and weight loss; large abdominal mass identified.
Day 2	Initial pelvic ultrasound: mass separate from uterus; ovaries initially reported as normal; small-volume free fluid.
Day 7	Clinical examination documented marked enlargement of the abdominal mass and cachectic appearance.
Day 8	CT abdomen/pelvis: giant complex abdominopelvic mass; hepatic abnormalities suspicious for metastatic disease; differential included choriocarcinoma and immature ovarian teratoma.
Day 8	Tumor markers: β-hCG 2 IU/L, AFP >60,500 µg/L, CA-125 322 U/mL, and CA19-9 1,314 U/mL.
Day 10	Subsequent ultrasound: bilateral cystic ovarian/adnexal masses with severe hepatomegaly and solid hepatic masses.
Day 13	Persistent anemia and abdominal discomfort; hemoglobin 8.5 g/dL after transfusion.
Day 15	Referral to Universitas Academic Hospital for further gynecological management.
Day 30	Right ovarian mass and attached fallopian tube received for pathological examination; specimen 3,697 g, 25 × 20.6 × 13.1 cm.
Day 38	Definitive pathology authorized: mixed germ cell tumor composed of mature cystic teratoma and YST; approximately 70% YST; no immature component; pT1a.
After surgery	Complete oncological follow-up was not available in the records reviewed.

## Discussion

Diagnostic challenge and radiological-pathological correlation

The central diagnostic finding was a YST-containing mixed germ cell tumor in a mass initially considered radiologically suggestive of immature teratoma or choriocarcinoma. MOGCTs are uncommon but are disproportionately represented among ovarian malignancies in adolescents and young adults [1-3]. The patient’s age, progressive abdominal enlargement, and very large complex ovarian mass were compatible with a germ cell tumor, but the extreme tumor size and reported hepatic abnormalities broadened the initial differential diagnosis.

The initial CT interpretation included choriocarcinoma and immature ovarian teratoma. This was understandable because MOGCTs can have overlapping complex solid-cystic appearances, and imaging may demonstrate necrosis, hemorrhage, local extension, peritoneal disease, or distant abnormalities [4]. However, the biochemical profile was more informative: β-hCG was only 2 IU/L, whereas AFP was >60,500 µg/L. The latter strongly supported a substantial YST component [1,4].

Large YSTs and immature teratomas can both demonstrate heterogeneous solid-cystic appearances. Immature teratomas may additionally contain variable amounts of fat and calcification, whereas YSTs frequently contain necrotic or hemorrhagic components [4,7]. Imaging can therefore narrow the differential and define tumor extent but may not reliably distinguish the components of a mixed germ cell tumor. In the present case, imaging favored immature teratoma, whereas histopathology identified mature cystic teratoma with a predominant YST component and specifically excluded an immature teratomatous component.

The principal radiological-pathological lesson is that a complex ovarian mass may suggest an immature teratoma without necessarily containing immature tissue. The definitive specimen contained mature tissues, including skin, adipose tissue, and cartilage, as well as keratin and hair within the cystic component, while the YST component comprised approximately 70% of the tumor volume. The extreme AFP elevation provided an important clue to the malignant germ cell component before definitive histological classification.

Histopathology and immunohistochemistry

The morphological findings, including Schiller-Duval bodies and microcystic and endodermal sinus patterns, supported the diagnosis of YST. SALL4 and cytokeratin positivity provided additional support when interpreted alongside the morphology and AFP elevation [1]. PLAP was also positive, while CD30 was negative. The immunophenotype should be considered as a panel rather than as a set of individually diagnostic stains. Histological examination was essential both for confirming the YST component and for establishing the mature nature of the teratomatous component.

Mixed germ cell tumors may contain combinations of dysgerminoma, YST, embryonal carcinoma, choriocarcinoma, and teratomatous elements. Goyal et al. described an unusual ovarian mixed germ cell tumor containing YST, mature teratomatous tissue, embryonal carcinoma, and trophoblastic differentiation [5]. The present case differs in composition, containing mature cystic teratoma and predominantly YST, and adds the context of a postpartum presentation with extreme tumor size.

AFP and clinical implications

AFP has an important role in the diagnosis and follow-up of ovarian YST. A systematic review and meta-analysis found that postoperative, rather than preoperative, AFP levels were prognostically informative, with persistently elevated postoperative AFP associated with poorer overall and relapse-free survival [8]. In the present case, postoperative AFP measurements were unavailable, so biochemical response and prognostic assessment could not be evaluated.

Management of MOGCTs differs from that of epithelial ovarian carcinoma. Fertility-sparing unilateral salpingo-oophorectomy is frequently appropriate in selected young patients when oncologically feasible, while platinum-based chemotherapy, particularly bleomycin, etoposide, and cisplatin (BEP), is an established component of treatment depending on histology and stage [3,9,10]. Although right salpingo-oophorectomy was documented in this case, the available records do not establish the extent of surgical staging or subsequent oncology treatment. Treatment adequacy and oncological outcome therefore cannot be assessed.

Postpartum presentation

The postpartum setting provides important clinical context but does not establish a causal relationship between pregnancy and tumor development. Miyamoto et al. reported a large ovarian YST diagnosed during pregnancy, treated with surgery and BEP chemotherapy, with no recurrence at 85 months [6]. That experience cannot be directly extrapolated to a patient diagnosed postpartum.

In this patient, progressive abdominal enlargement persisted for approximately six months after delivery and was accompanied by pain, weight loss, amenorrhea, and a palpable mass. These features warranted investigation for structural pathology. The early ultrasound findings were nonspecific, with the mass described as separate from the uterus and the ovaries initially considered normal. A subsequent ultrasound described bilateral cystic adnexal masses, while CT demonstrated the extensive abdominopelvic mass. Because the original ultrasound images were unavailable, the discrepancy in adnexal localization cannot be independently reconciled and should not be interpreted as proof of interval development or progression. The records do not establish whether attribution of symptoms to postpartum change delayed investigation.

Hepatic findings and staging

The hepatic findings are particularly important when interpreting stage. The pathology report assigned pT1a to the primary ovarian tumor and explicitly noted that the clinical history of liver metastasis was not histologically confirmed. The pT1a category should therefore not be treated as proof of stage I disease. Conversely, the absence of histological confirmation does not exclude hepatic metastases. The hepatic lesions are best described as radiologically suspicious, with the overall stage left unresolved because the available records do not establish their nature.

This distinction between radiological suspicion, pathological primary-tumor category, and overall clinical stage is important in case reporting. It prevents an uncertain imaging finding from being presented as definitive metastatic disease while also avoiding the opposite error of assuming that lack of biopsy proof excludes metastasis.

Limitations

The principal limitation is the absence of postoperative treatment and outcome data, including serial AFP measurements. Detailed antenatal records and original pregnancy ultrasound examinations were unavailable, so the course of the pregnancy and antenatal adnexal imaging cannot be further characterized. Original ultrasound images, DICOM CT data, gross specimen photographs, and histopathological photomicrographs were also unavailable. The CT screenshots illustrate tumor bulk but do not permit confident independent assessment of the reported hepatic lesions or precise imaging-pathology mapping of individual tumor components. The discrepancy between the initial ultrasound reporting apparently normal ovaries and the later ultrasound describing bilateral cystic adnexal masses cannot be resolved from the available retrospective records. Tumor markers were documented on D8 and a post-transfusion hemoglobin of 8.5 g/dL on D13, but exact sampling times were unavailable for some other laboratory measurements. Reference intervals for CA-125 and CA19-9 were unavailable in the supplied laboratory report. The hepatic lesions remained radiologically suspicious rather than histologically confirmed metastases, and pT1a refers to the primary ovarian tumor rather than establishing the overall clinical stage. Postoperative oncology records from Universitas Academic Hospital, including systemic chemotherapy details, postoperative AFP values, evolution of the hepatic lesions, and long-term outcome, were unavailable; no treatment-response or prognostic conclusions are therefore inferred.

## Conclusions

Progressive postpartum abdominal enlargement accompanied by pain, weight loss, amenorrhea, or a palpable mass warrants investigation for underlying pathology. In this case, marked AFP elevation supported a YST component, while histopathology established a mixed tumor containing mature cystic teratoma and predominantly YST without an immature teratomatous component. The case highlights the need to integrate imaging, tumor markers, and histopathology because imaging alone cannot determine the precise composition of a mixed germ cell tumor. The hepatic lesions remained radiologically suspicious rather than proven metastases, and the reported pT1a category describes the primary ovarian tumor rather than establishing the overall stage. Postoperative treatment, AFP response, and long-term outcome were unavailable and are not inferred.
